# Nanoencapsulated capsaicin changes migration behavior and morphology of madin darby canine kidney cell monolayers

**DOI:** 10.1371/journal.pone.0187497

**Published:** 2017-11-06

**Authors:** Mathias Kaiser, Luisa Pohl, Steffi Ketelhut, Lena Kastl, Christian Gorzelanny, Martin Götte, Jürgen Schnekenburger, Francisco M. Goycoolea, Björn Kemper

**Affiliations:** 1 Institute of Plant Biology and Biotechnology (IBBP), Westfälische Wilhelms-Universität Münster, Schlossgarten 3, Münster, Germany; 2 Biomedical Technology Center of the Medical Faculty, Westfälische Wilhelms-Universität Münster, Mendelstraße 17, Münster, Germany; 3 Experimental Dermatology, Department of Dermatology, Medical Faculty Mannheim, Heidelberg University, Theodor-Kutzer-Ufer 1–3, Mannheim, Germany; 4 Department of Gynecology and Obstetrics, Westfälische Wilhelms-Universität Münster, Albert-Schweitzer-Campus 1, Münster, Germany; 5 School of Food Science & Nutrition, University of Leeds, Leeds, United Kingdom; Pennsylvania State Hershey College of Medicine, UNITED STATES

## Abstract

We have developed a drug delivery nanosystem based on chitosan and capsaicin. Both substances have a wide range of biological activities. We investigated the nanosystem’s influence on migration and morphology of Madin Darby canine kidney (MDCK-C7) epithelial cells in comparison to the capsaicin-free nanoformulation, free capsaicin, and control cells. For minimally-invasive quantification of cell migration, we applied label-free digital holographic microscopy (DHM) and single-cell tracking. Moreover, quantitative DHM phase images were used as novel stain-free assay to quantify the temporal course of global cellular morphology changes in confluent cell layers. Cytoskeleton alterations and tight junction protein redistributions were complementary analyzed by fluorescence microscopy. Calcium influx measurements were conducted to characterize the influence of the nanoformulations and capsaicin on ion channel activities. We found that both, capsaicin-loaded and unloaded chitosan nanocapsules, and also free capsaicin, have a significant impact on directed cell migration and cellular motility. Increase of velocity and directionality of cell migration correlates with changes in the cell layer surface roughness, tight junction integrity and cytoskeleton alterations. Calcium influx into cells occurred only after nanoformulation treatment but not upon addition of free capsaicin. Our results pave the way for further studies on the biological significance of these findings and potential biomedical applications, e.g. as drug and gene carriers.

## Introduction

In drug delivery the utilization of nanocarrier systems provides increased bioavailability as well as the generation of specific targeted effects and for this reason is highly in focus of current research [1]. During the last decades, many devices for drug delivery and diagnostics were developed. Most of these approaches consist of synthetic polymers and metallic nanoparticles [2–4] but only very few of these systems are based on naturally derived biopolymers like, for example, proteins and polysaccharides [5,6]. Recently, biopolymer-based approaches for drug transport vehicles have emerged. Such biomaterials share similar building blocks with structures in living organisms like bone, shells, hair, and plant fibers [7] and are organized in likewise hierarchical structures and thus promise a higher biocompatibility compared to their synthetic counterparts. Bioinspired or biomimetic nanobiomaterials are therefore believed to be promising key candidates in the development of novel approaches for diagnostics and improved treatment of diseases [8].

Alonso et al. advanced a method to obtain colloidal nanocapsules based on solvent displacement (or spontaneous emulsification) [9]. In further studies, this approach has been demonstrated to be an effective platform for the small lipophilic or macromolecular hydrophilic drugs and vaccines’ delivery [9–16]. In particular, oil core-shell nanocapsules comprising natural compounds which are stabilized by lecithin were identified to be attractive candidates [17–19]. To generate such nanosystems, organic and aqueous liquid phases of the source materials only need to be gently mixed and capsules form spontaneously without further need of stirring or emulsification [9].

We have developed a nanocapsule drug delivery system based on the biopolymer chitosan which is known to increase paracellular permeability through epithelial barriers. Chitosan, a family of cationic natural aminopolysaccharides, is known for its various interactions with biological barriers, like mucoadhesive properties [20], the ability to reversibly open cellular tight junctions (TJs) [21] as well as for its high biocompatibility and biodegradability [22,23]. Several studies have addressed the mechanisms of chitosan TJs opening in mammalian epithelia in cell cultures, [21,24–29] *ex vivo* as well as in animal models [28]. Several suggestions have been advanced to explain these effects. The early studies by Schipper et al. [24] and more recent ones [30] convene in that chitosan redistributes ZO-1 and cytoskeletal F-actin [24]. These effects were found to be mediated by chitosan’s positive charges in glucosamine residues [24]. Afterwards, it has been proposed that the overriding mechanism is due to claudin-4 (CLDN4) redistribution [21]. Recently, it has also been suggested that the mechanism of the activity of chitosan of opening tight junctions underlays on redistribution of JAM-1 (junction adhesion molecule) [28]. The influence of chitosan’s N-acetylation and molecular weight on cell permeability has also been addressed [26]. Chitosan’s permeabilizing activity for bioactive macromolecules due to the reversible opening of TJs has also been found to persist in nanoformulations such as nanoparticles and nanocapsules [12,17,31–35].

In this work, we utilized chitosan as a coating for capsaicin-loaded nanocapsules which was used as model for lipophilic drugs. Capsaicin is a natural compound occurring in hot chili peppers that is well-known for its pungency [36]. We chose the substance due to its various biological activities and its widespread usage in traditional and scientific medicine to treat various diseases [37], which includes the therapy of chronic pain, obesity [38], and urologic disorders [39] as well as body temperature regulation [37]. One of the earliest discovered action modes of capsaicin is its interaction with the transient receptor potential vanilloid 1 (TRPV1), a nonselective cation channel which is involved in the detection of body temperature and heat nociception [40]. Recently, it was reported that capsaicin is able to open reversibly the TJs of epithelial cells [41]. It was also shown that the calcium influx into cells is involved in the capsaicin-mediated TJs opening process [42], and can have an influence on cellular migration [43] via the stimulation of the TRPV1 receptor. For MDCK and Caco-2 cells, it was demonstrated that cofilin-actin cytoskeletal dynamics and a decreased level of occludin are responsible for the reversible opening effect [41,42,44–48]. In these studies the distributions of several TJs proteins including occudin, claudin, ZO-1 and others were investigated.

In a previous study, we incubated Madin Darby canine kidney (MDCK)-C7 cells, an *in vitro* model for an epithelial barrier, with encapsulated and free capsaicin [17]. We found chitosan-based capsules were internalized by the cells and that the encapsulation of capsaicin with chitosan significantly attenuated cytotoxicity and modulated the trans-epithelial electrical resistance (TEER), compared with free capsaicin. This makes our system a potential co-carrier candidate for the enhancement of bioavailability of poorly absorbable macromolecular drugs. We also observed that stimulation of MDCK-C7 cells with capsaicin-loaded chitosan-based nanocapsules induces reversible disruptions of the cell-cell contacts and concomitant changes of the cell morphology.

The aim of this study is to investigate the influence of nanoencapsulated and free capsaicin on cell migration compared to its free form which, to the best of our knowledge, has not been addressed before, and to analyze its impact on cellular morphology.

To this end, we developed a novel microscopy assay based on digital holographic microscopy (DHM) [49–51], an interferometry-based variant of quantitative phase microscopy (QPM) [52], for minimally-invasive time-lapse live cell observation. QPM provides label-free imaging by detection of optical path length changes. In DHM the sample is illuminated in transmission. Thus, only low light intensities are required which minimizes the interaction with living cell cultures and allows continuous long-term monitoring without the need of predefined endpoints. The resulting sequences of quantitative DHM phase contrast images were analyzed by single-cell tracking to quantify cell direction and speed. Moreover, by evaluation of QPM-based accessed optical path length changes and utilizing novel image analysis procedures, global morphology changes of confluent MDCK-C7 cell monolayers were quantified. In complementary experiments with fluorescence microscopy we analyzed the dynamics of calcium fluxes inside the cells as well as rearrangements of TJs proteins and the cytoskeleton to confirm changes in cellular morphology. Our results reveal that capsaicin-loaded and unloaded chitosan nanocapsules as well as free capsaicin significantly influences directed cell migration and cellular motility. These effects are accompanied by changes in the global cell layer surface roughness. The observed tight junction integrity and actin cytoskeleton changes are in agreement with previous studies.

## Materials and methods

### Cell culture

Madin Darby canine kidney (MDCK)-C7 cells were cultured using MEM supplemented with 10% of fetal bovine serum, 1% of L-glutamine (200 mM) and 1% of penicillin-streptomycin (10000 units penicillin, 10,000 units streptomycin in 0.9% NaCl) in 75 cm^2^ flasks. The cultures were kept in an incubator set to 5% of CO_2_ and 37°C (Sanyo MCO-19AIC, Panasonic Biomedical Sales Europe BV., AZ Etten Leur, Netherlands). For all experiments, the passages 41 to 47 were used. After forming a confluent monolayer, the cells were rinsed with 10 ml of Phosphate buffered saline (PBS) and trypsinized with 10 ml of trypsin buffer (0.05% trypsin EDTA). The trypsin buffer was diluted after detachment with 10 ml of cell culture medium. The cells were centrifuged at 300 rcf for 5 min in a falcon tube (Centrifuge: Rotina 420 R, Hettich GmbH, Tuttlingen, Germany). Supernatant was removed, and the cell pellet was resuspended in 1 ml of cell culture medium. For counting, 10 μl of cell suspension was diluted with 90 μl of trypan blue and transferred to an improved Neubauer chamber. Afterwards, the cells were used for experiments. For subculture, the cells were split in the ratio of 1:10. All experiments were performed independently in triplicates on three different days.

### Preparation of nanoformulations

The chitosan-coated nanocapsules were prepared as previously described with modifications [17,19]. Briefly, for the *in vitro* experiments, 400 μl of a 100 mg/ml ethanolic lecithin solution (Epikuron 145 V, Cargill texturing solutions Deutschland GmbH &Co. KG, Hamburg, Germany) was mixed with 530 μl of the capsaicin stock solution (24 mg/ml). This was supplemented with 125 μl Miglyol 812 N (Sasol GmbH, Witten, Germany) and 9.5 ml ethanol. The organic solution was immediately poured into 20 ml chitosan in the aqueous phase (0.5 mg/ml in 5% stoichiometric excess of HCl). The milky mixture was concentrated in a rotavapor (Büchi R-210, Büchi Labortechnik GmbH, Essen, Germany) at 50°C until 3.5–4.0 ml remained and the volume was topped up to 4.0 ml with milliQ water if necessary to yield a final capsaicin concentration of ~10 mM. The nanoemulsions were prepared using the same procedure but without including chitosan. Unloaded nanocapsules and nanoemulsions were prepared by replacing the capsaicin solution with ethanol. The physical characteristics of the nanocapsules have been documented in a previous study [17]. Briefly, the size of the formulations was around 200 nm, the zeta potential was highly positive (~ +60 mV) and the polydispersity index had a value of ~ 0.2. These values were obtained by dynamic light scattering.

### Digital holographic microscopy (DHM)

For quantitative live cell imaging DHM [49–51], a variant of quantitative phase microscopy (QPM) [52] was applied. Compared to state-of-the-art Zernike phase microscopy [53] and differential interference contrast (DIC) [54], QPM provides high-resolution label-free imaging by detection of optical path length changes without halo and shading artifacts [49]. As the object is illuminated in transmission only low light intensities are required. Thus, QPM minimizes the interaction with the sample which is highly beneficial for minimally-invasive long-term monitoring of sensitive biological specimens [55,56]. For investigations with DHM cells were seeded in Petri dishes with glass lid (ibidi μ-Dish with glass lid, ibidi GmbH, Munich, Germany) in MEM at a density of 2.1 x 10^5^ cells/dish and were allowed to attach overnight. The following day the medium was replaced with MEM lacking supplements but containing 50 μM of capsaicin or the nanoformulations with the same capsaicin concentration (equaling 2.35 mg/ml of the capsule formulation) in 20 mM HEPES (4-(2-hydroxyethyl)-1-piperazineethanesulfonic acid) buffer. Quantitative label-free DHM imaging of MDCK cells was carried out using an inverted microscope (iMIC, Till Photonics, Gräfelfing, Germany) with an attached DHM module [57], and an incubator (Solent Scientific Ltd., Segensworth, UK) to ensure temperature stability at 37°C. The coherent light source for the capture of digital holograms was a frequency-doubled neodymium-doped yttrium aluminum garnet (Nd:YAG) laser (Compass 315 M-100, Coherent, Lübeck, Germany, λ = 532 nm). Digital off-axis holograms of confluent mono cell layers were recorded continuously every 10 min using a 20x microscope lens (Zeiss LD Acroplan 20x/0.4 Korr). From the digitally-captured holograms quantitative phase images were reconstructed with custom-built software based on spatial phase shifting as previously described [58,59]. Three independent time-lapse measurements were taken in each experiment. Complementary cell viability assays simulating the cell culture conditions during DHM analysis (including HEPES buffer but without a CO_2_-enriched atmosphere) showed a cytotoxic effect of capsaicin starting at a concentration of ~200 μM (see Supporting Information). We, therefore, chose a concentration of 50 μM capsaicin for the DHM experiments.

### Quantification of cell migration

For each time lapse measurement, ten cells were tracked manually in the resulting series of DHM quantitative phase images by using the Tracking Plugin of the free software ImageJ (National Institutes of Health [NIH], Bethesda, MD, http://rsb.info.nih.gov/ij/). From the resulting migration trajectories, velocity and directionality (**Eq 1**) were calculated and visualized as plots with the freely available Chemotaxis and Migration Tool (ibidi GmbH, Munich, Germany).

$$\mathrm{directionality}=\frac{\mathrm{euclidean}\;\mathrm{distance}}{\mathrm{accumulated}\;\mathrm{distance}}$$ (1)

### Quantification of morphology changes using DHM quantitative phase images

In contrast to confocal laser scan microscopy (CLSM) [60,61] and related fluorescence-based techniques [62,63] that provide molecule specific signals based on light intensity acquisition, DHM accesses integral phase information about optical path length changes at low light intensities which is related to the cellular thickness and the spatial distribution of intracellular solute concentrations. The resulting spatial distribution of the cell induced DHM phase contrast Δ*φ* depends on the cell thickness *d*, the integral cellular refractive index *n*_cell_, the refractive index *n*_medium_ of the cell culture medium [49,59] and the wavelength *λ* of the laser light used in the DHM system:

$$\Delta \varphi \left(x,z\right)=\frac{2\pi }{\lambda }(n_{\mathrm{cell}}-n_{\mathrm{medium}})d\left(x,z\right).$$ (2)

**Fig 1:**
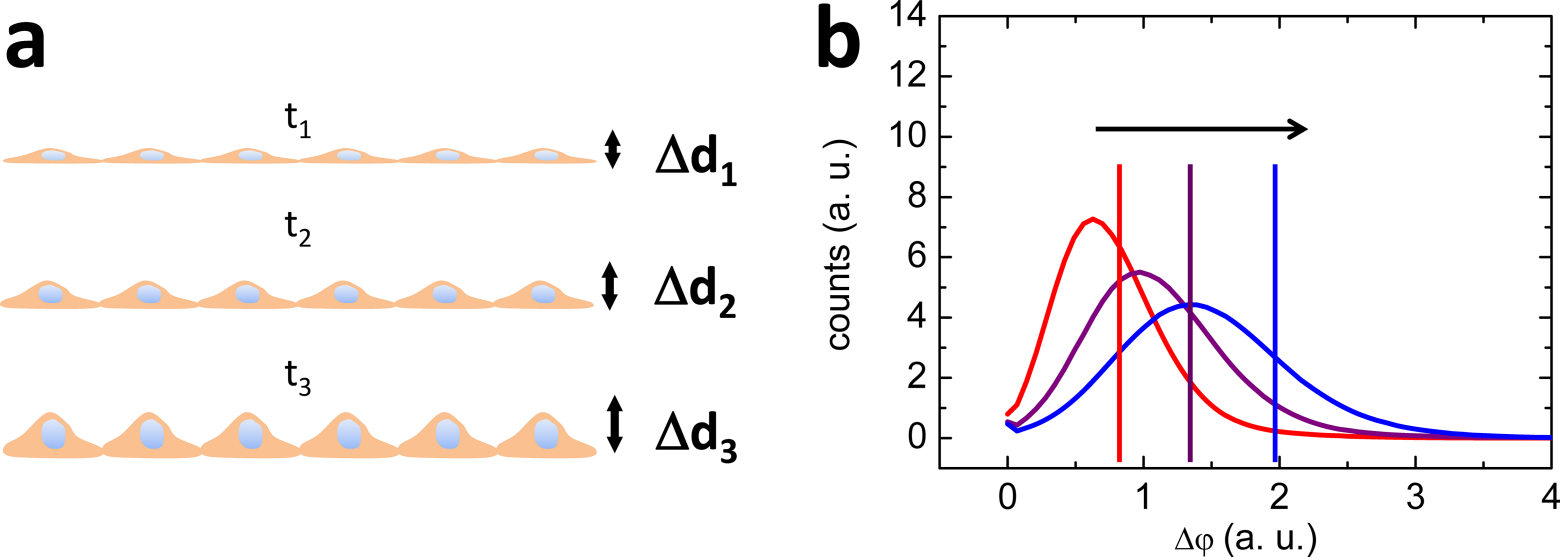
Analysis of global morphology changes in confluent cell layers. Analysis of global morphology changes in confluent cell layers by histogram-based evaluation of quantitative DHM phase contrast images at different time points (red: t_1_, purple: t_2_, blue: t_3;_ t_1_ < t_2_ < t_3_). An increase of the cell layer surface roughness Δ*d* (a) causes a shift of the histogram (b) and the average phase contrast $\Delta \bar{\varphi }$ (vertical lines in (b)) towards higher phase values.

For a confluent cell layer and assuming the parameters *n*_cell_, *n*_medium_ and *λ* to be constant during the experiment, the average phase contrast $\Delta \bar{\varphi }$ is proportional to the average relative surface roughness $\Delta \bar{d}$

$$\Delta \bar{\varphi }=\frac{2\pi }{\lambda }(n_{\mathrm{cell}}-n_{\mathrm{medium}})\Delta \bar{d}$$ (3)

and thus, quantifies global cell morphology changes (for illustration see **Fig 1**) after treatment with capsaicin and the nanoformulations. To visualize and quantify such cellular morphology changes, the histograms of quantitative phase contrast images of confluent cell layers were plotted, and the average phase contrast $\Delta \bar{\varphi }$ was calculated at certain time points after the addition of the respective agent (capsaicin or nanoformulations). Therefore, in a first step, the quantitative phase images resulting from DHM time-lapse observations as shown in video **S1 Movie** were corrected for background fluctuations using the "subtract background" option of ImageJ. Then, the background corrected phase images were analyzed at certain time points. MDCK-C7 cells migrated very fast within the cell mono layer during the observation periods in the observed field of view (for illustration see video **S1 Movie** in the Supporting Information). Thus, to compensate for this effect, 4 time-lapse phase images prior and 5 phase images after each time point were considered using the “stack” plugin of ImageJ. The resulting averaged histograms of the phase images around each time point were plotted using Prism v6.0c (GraphPad Software Inc., La Jolla, USA). Note that the averaging process of the phase data during the histogram generation also reduces the influence of the temporally randomly fluctuating disturbances in the quantitative DHM phase images as visible in video **S1 Movie** which are caused by the coherence properties of the applied laser light and fluctuating small particles in the cell culture medium. In addition, for each chosen time point the averaged phase contrast was calculated:

$$\Delta \bar{\varphi }=\frac{1}{n_{x}\times n_{y}}\sum_{i,j=1}^{n_{x},n_{y}}\Delta \varphi_{i,j}.$$ (4)

In Eq (4), the parameters *n*_*x*_ and *n*_*y*_ denote number of image pixels in x and y direction and Δ*φ*_*i*,*j*_ denotes the phase values of the image pixels.

### Calcium influx measurements

For calcium influx imaging, cells were seeded to form a confluent monolayer in an eight-well microscopy slide (μ-Slide 8 Well ibiTreat, ibidi GmbH, Munich, Germany) and were allowed to attach to the surface overnight. After a single washing step with supplement free medium in each well the cells were incubated for 20 min 100 μl of cell culture medium containing Fluo-4 (1 μl of 5 mM stock solution in 1 ml). Then cells were rinsed with HEPES-buffered Ringer’s solution (10 mM HEPES, 5 mM glucose, 1 mM CaCl_2_, 1 mM MgCl_2_, 5 mM KCl, 140 mM NaCl) to remove the excess dye. Finally, 200 μl of HEPES buffer was filled in each well, and the slide was immediately used for the experiment. Imaging was performed using structured illumination fluorescence microscopy (SIFM) based on an inverted fluorescence microscope (Zeiss AxioObserver.Z1, Goettingen, Germany) equipped with a structured illumination module (ApoTome, Jena, Germany). Fluorescence images were acquired at a rate of 0.88 Hz. After recording 20 frames, 100 μl of a sample containing 375 μM of the nanoformulation was added with a pipette to reach an overall concentration of 125 μM and the time-lapse experiment was continued for further 5 minutes. All images series were acquired with the same adjustments for excitation light intensity and camera exposure times. The resulting fluorescence images were analyzed using ImageJ. First, the background intensity was subtracted using the “rolling ball algorithm” (ball radius = 500 pixels). Then the brightness of all series was normalized, and the brightness value prior the addition of the treatments was defined as zero. The temporal development of the average image brightness was plotted using Prism version 6.0c (GraphPad Software Inc., La Jolla, USA).

### Fluorescence microscopy of actin and ZO-1

For immunofluorescence staining MDCK-C7 cells were seeded on glass cover slips and cultivated until confluency (3 days). Cells were treated with nanocapsules (1:40) or capsaicin (125μM) for 6 h and fixed with methanol for 20 minutes at -20°C.

For analysis of the actin distribution cell membranes were permeabilized with 0.2% Triton-X-100 in PBS (Sigma) for 5 min and incubated with 130 nM Alexa Fluor 488 labeled Phalloidin (Invitrogen) for 45 minutes. Nuclei were stained with 0.5 μM 4’,6-Diamidino-2-phenylindole dihydrochloride (DAPI, Sigma-Aldrich) for 10s. Cover slips with fluorescence labeled cells were embedded in Aqua Poly/Mount (Polysciences) and imaged with an Olympus IX-81 inverted fluorescence microscope via a 60x UPLSAPO oil objective (Olympus) and a CCD camera (F-View, Olympus Soft Imaging Muenster, Germany).

For fluorescence imaging of ZO-1, cells were washed with HEPES-buffered Ringer solution ((10 mM HEPES, 5 mM glucose, 1 mM CaCl_2_, 1 mM MgCl_2_, 5 mM KCl, 140 mM NaCl) and blocked with 2% BSA in incubation buffer (HEPES-buffered Ringer solution, 0.3% Triton-X 100, 0.2% BSA). The primary antibody directed against zonula occludens-1 (617300, invitrogen) was diluted in incubation buffer (1:150) and co-incubated with cells for 4 h at room temperature. The secondary FITC-labeled antibody, diluted in incubation buffer (1:200) (BD Pharmingen. 554020), was applied for one hour at room temperature. Cell nuclei were stained with DAPI. Finally, glass cover slips were mounted on a glass object slide using DABCO-Mowiol. Samples were analyzed with an inverted fluorescence microscope (Zeiss Axio Observer.Z1, Göttingen, Germany) and the ZEN software (Zeiss, Germany).

In order to achieve maximum quality of merged images fluorescence detection channels have been focused separately if required.

### Statistical analysis

Statistical analysis of data was carried out using Prism v6.0c (GraphPad Software Inc., La Jolla, USA). All experiments were statistically analyzed using non-parametric tests. For all experiments, the Kruskal-Wallis test in combination with Dunn's multiple comparison test was applied.

## Results

### Nanoformulations and capsaicin influence the migration of MDCK-C7 cells

To investigate the migration behavior of the MDCK-C7 cells after incubation with sub-cytotoxic concentrations of capsaicin-loaded nanocapsules, unloaded nanoformulation, free capsaicin and control treatments, we carried out time-lapse measurements using label-free digital holographic microscopy (DHM). To this end, confluent cell monolayers were observed for 18 h by taking digital holograms every ten minutes. Video **S1 Movie** (Supporting Information, images were contrast enhanced for better visualization) of the obtained quantitative phase images illustrates the swarm-like migration behavior of the cells and shows representative measurement results for each treatment. The control cells migrated slowly in a random movement pattern while the cell contours were not clearly visible. Cells treated with free capsaicin moved very similar but, in contrast, the cell boundaries were visible during the whole experiment and after ~8 h, formation of gaps between the cells was observed. For the cells treated with unloaded and capsaicin-loaded nanocapsules the swarm-like migration behavior altered to highly directed movements. Moreover, the migration speed of the cells treated with unloaded nanocapsules significantly accelerated in comparison to the control, while the cell outlines were more clearly distinct. For capsaicin-loaded nanocapsules, in analogy to the treatment with free capsaicin, after ~13 h, a formation of gaps between the cells was observed.

**Fig 2:**
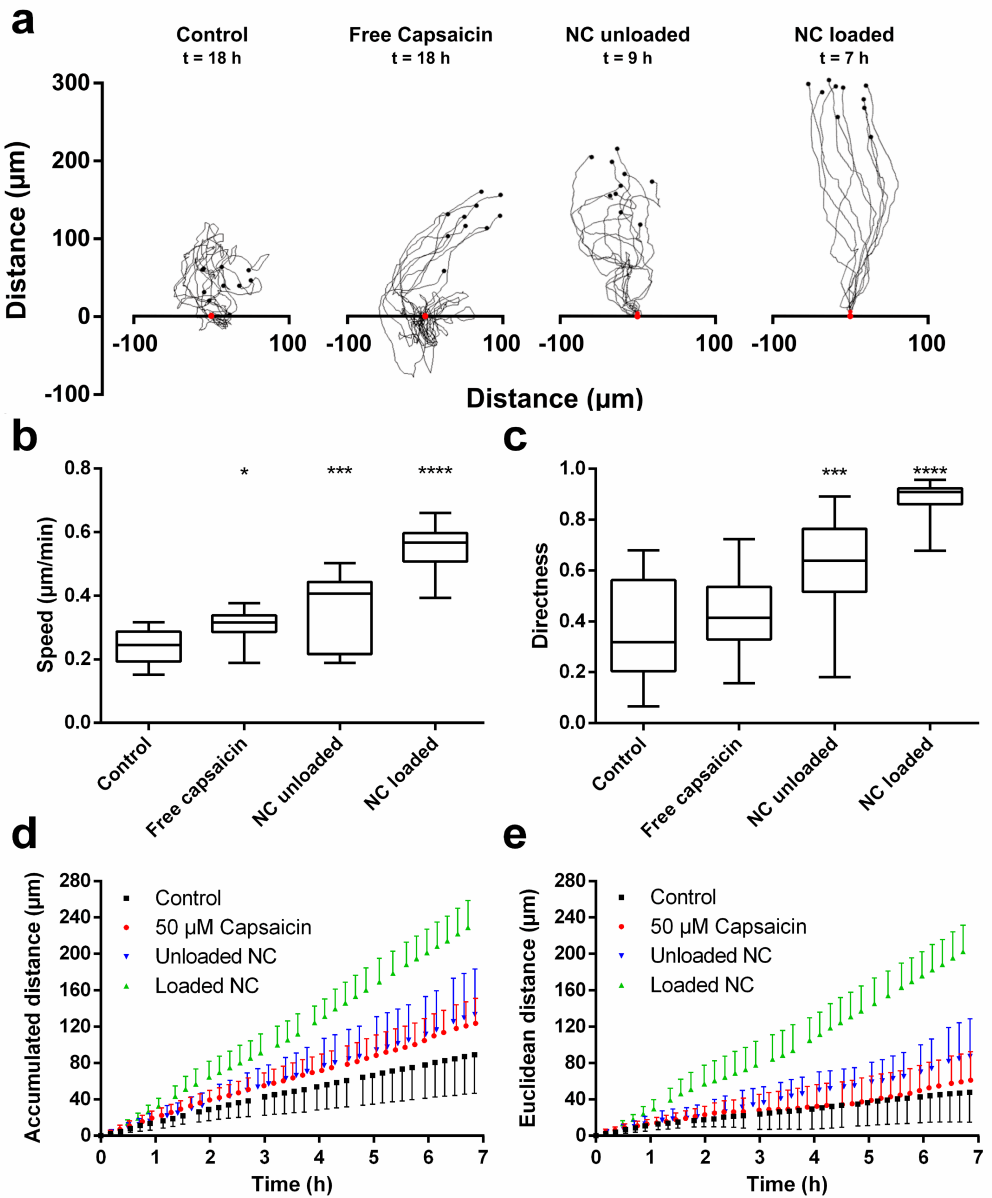
Migration patterns of MDCK-C7 cells. Migration patterns of MDCK-C7 cells after treatment with nanocapsules and free capsaicin: (a) Representative migration tracks. End times of the tracking experiment are given below the headlines as the cells moved out of the observed field of view at different time points due to different migration speeds. (b) Migration speed as an end point measurement. (c) Directness as an end point measurement. (d) Accumulated distance over time for all the treatments. (e) Euclidean distance over time for all the treatments. Mean values ± SD. Statistical test: Kruskal-Wallis test (n = 3; 10 cells per measurement), * p < 0.05, *** p < 0.001, **** p < 0.0001). NC: nanocapsules.

**Fig 2**summarizes the results from the quantitative evaluation of the DHM phase contrast images by single-cell tracking. In **Fig 2A** representative 2D single-cell tracks are plotted. All tracks are arranged by adjusting their origin in the same starting point which is marked with a red dot at the respective x-axis. The lines represent the migration trajectories of each analyzed cell while the black dots indicate the final cell position at the end of each track. Due to their high migration velocities, the cells treated with the nanocapsules already left the field of view much earlier than the cells stimulated with the other treatments. Thus, the time durations of each tracking experiment, given above each plot, are not identical for the different treatments. The trajectories of the control cells show, in correspondence with video **S1 Movie** (Supporting Information), a mainly non-directed random pattern with an average maximum migration distance of ~100 μm from their initial position. The addition of 50 μM free capsaicin to the cell culture medium changed the initial random movements of the cells to a more straight migration. The tracks from the cells treated with unloaded chitosan nanocapsules show a further increase of directed movements. Treatment with capsaicin-loaded nanocapsules led even to almost linear cell migration trajectories and average maximum migration distances of about ~300 μm from the origin, which represents about the 3-fold distance compared to the control. Due to the high migration velocity some of the cells left the field of view prior the end of the time-lapse observation experiment. Thus, the further quantitative evaluation of the migration trajectories was limited to the first 7 hours of the experimental period. **Fig 2B** summarizes the retrieved average migration velocities from three independent experiments for each cell treatment and control cells. A statistically significant velocity increase was found for all treatments compared to the control (capsaicin *p* < 0.5; unloaded nanocapsules *p* < 0.001; capsaicin-loaded loaded nanocapsules *p* < 0.0001, Kruskal-Wallis test). **Fig 2C** shows the averaged directionality derived from the migration trajectories. For all treatments, the directionality is found to be higher than for the control. This is in agreement with the appearance of the migration tracks shown in **Fig 2A**. The two nanoformulations induced a significant increase of the directionality values compared to the control (unloaded nanocapsules *p* < 0.001, loaded nanocapsules *p* < 0.0001, Kruskal-Wallis test) while for free capsaicin, however, the observed increase was non-significant (*p* > 0.5, Kruskal-Wallis test). **Fig 2D** depicts the temporal dependency of the averaged accumulated migration distance. Note the almost linear trends of all curves. **Fig 2E** shows the corresponding data for the Euclidean distance, between the starting point and the end point of the single-cell migration trajectories. In agreement with the results obtained for the accumulated distance for all treatments, highly linear trends are also observed for the Euclidean distance. Notice that the curves of the cells treated with capsaicin-loaded nanocapsules show the highest gradient. In contrast, only a slight difference was found between the data from unloaded nanocaspules and free capsaicin whereas the curves of the control cells showed the smallest slope.

### Nanoformulations and capsaicin cause cell layer morphology changes

**Fig 3:**
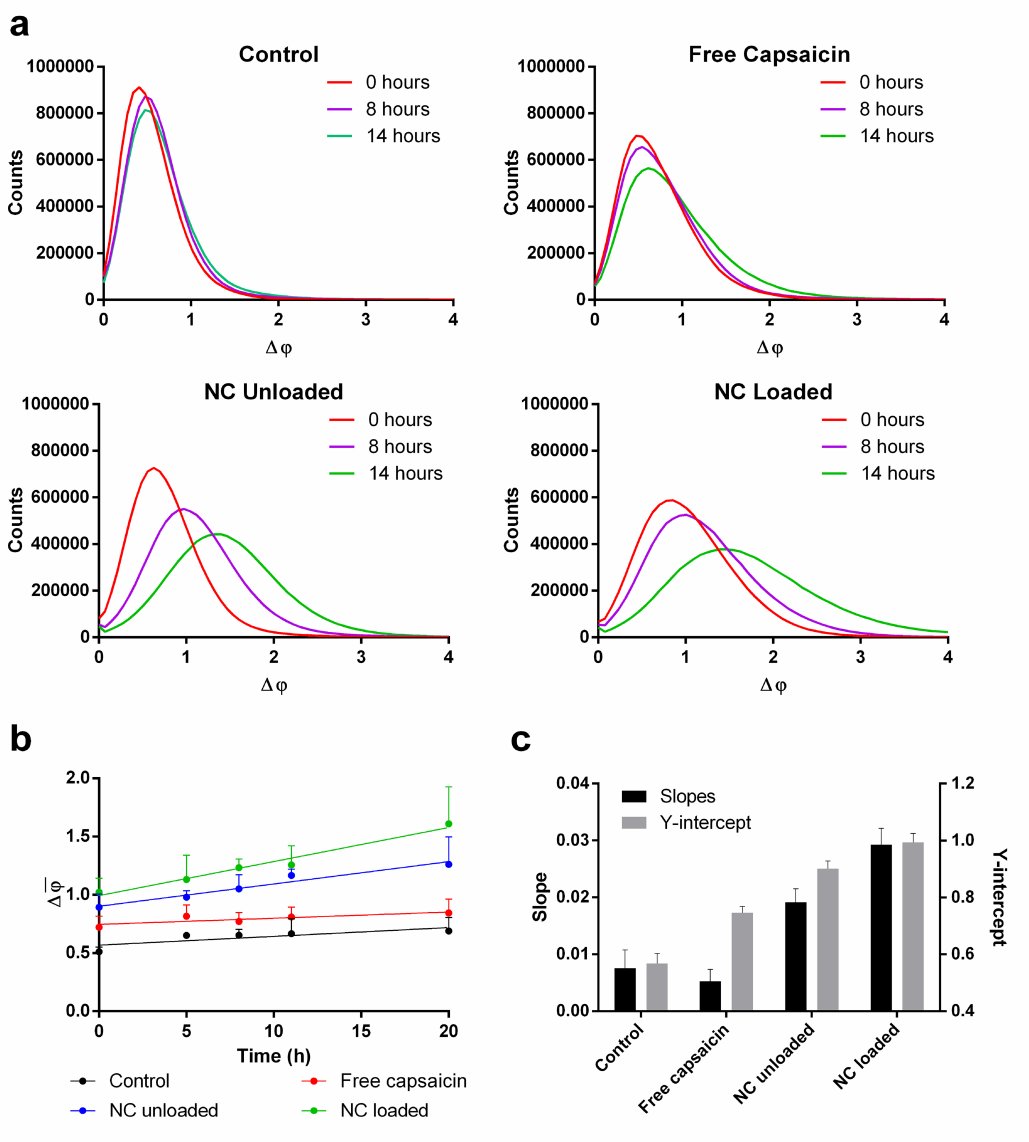
Analysis of cell morphology by digital holographic microscopy. Analysis of cell morphology by evaluation of quantitative phase images retrieved with digital holographic microscopy: (a) Histograms of quantitative DHM phase contrast images at different time points for control cells and cells treated with capsaicin and the different nanoformulations. (b) Average phase contrast $\Delta \bar{\varphi }$ for all treatments plotted over time with a linear fit. (c) Slopes and Y-intercepts retrieved by the linear fits from (b). Mean values ± SD (*n = 3*).

To quantify the global morphological changes induced by the different treatments, the confluent cell layers were analyzed by histogram-based evaluation of quantitative DHM phase contrast images (see detailed description in the materials and methods section). **Fig 3**summarizes the results. In **Fig 3A** the histograms of quantitative phase images for a representative measurement at t = 0 h, 8 h and 14 h are plotted. The parameter Δ*φ* detects the spatially dependent optical path length delays that are related to the thickness changes of individual cells in the confluent monolayers. Thus, the histograms of the phase values quantify global alterations of the cell layer surface roughness during the time course of the experiments (see materials and methods section). For control cells, the histograms remained nearly unchanged during the experimental period. This indicates no changes of the average cell layer surface roughness. The treatment with capsaicin caused a shift of the histograms towards higher phase values. Also, a broadening of the histograms was observed. Both parameter changes are indicative for a higher cell layer surface roughness than found for the control cells. These effects were even more pronounced for the treatments with capsaicin-loaded and unloaded nanoformulations. The histograms shifted further to the right and became broader and flatter compared to capsaicin-treated cells and control cells. In **Fig 3B** the average phase values corresponding to the histogram data in **Fig 3A** are plotted for certain time points. For the control and all treatments, linear trends are observed. **Fig 3C** shows the slopes and Y-axis intercepts retrieved from linear fits to the temporal development of the average phase contrast in **Fig 3B**. For free capsaicin, the slope was slightly lower than the control. In agreement with the data in **Fig 3B** the treatment with unloaded nanocapsules resulted in a higher slope in comparison to free capsaicin and the control, while the maximal slope was found for the capsaicin-loaded nanocapsules. The corresponding abscissa values showed the same ascending trend. This result indicates that cell layer surface roughness changes are already induced within the range of several minutes after the addition of the different substances to the MDCK cells prior the start of the DHM time-lapse observation.

### Nanoformulations trigger calcium fluxes in the cells

**Fig 4:**
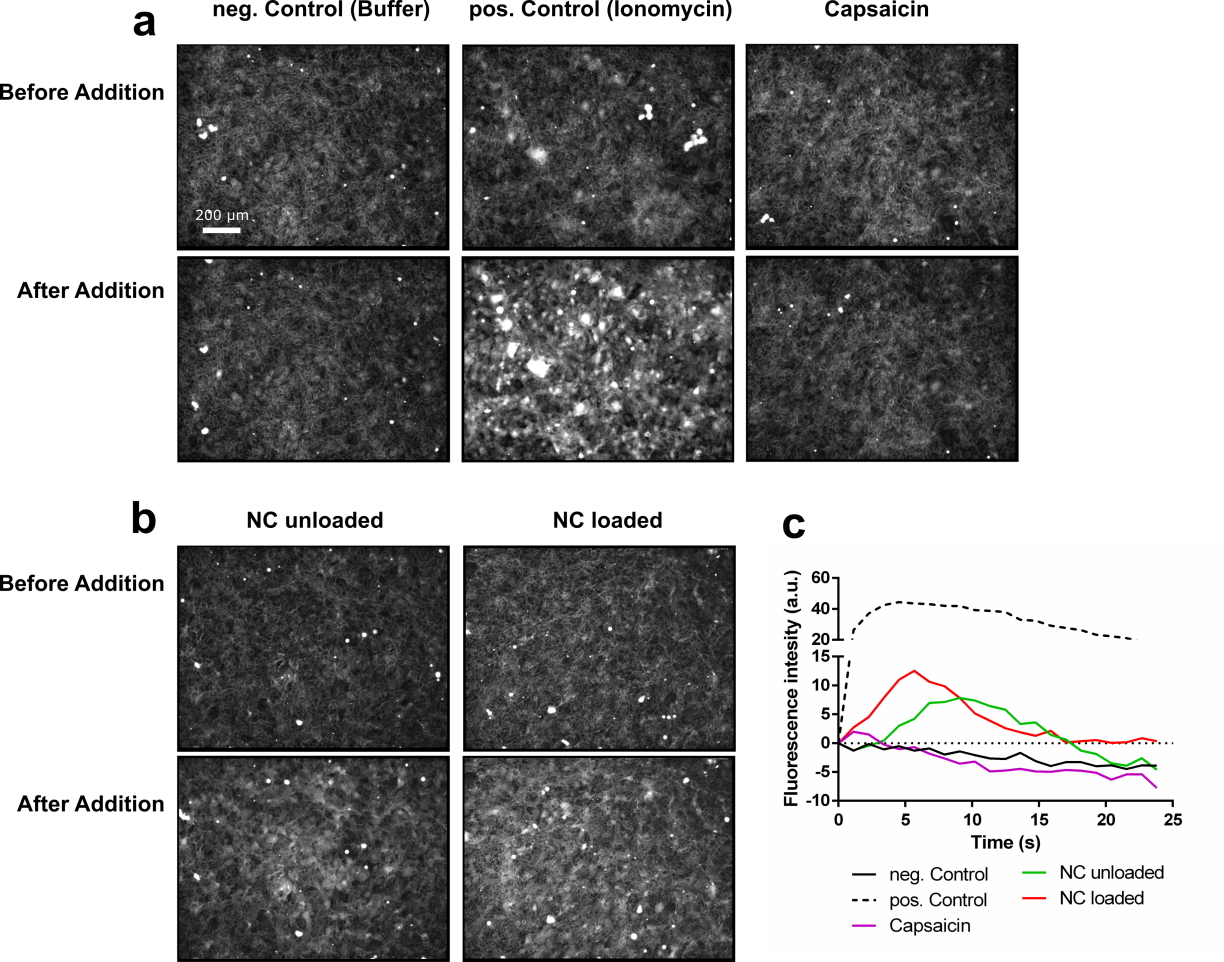
Calcium flux experiments. Calcium flux experiments using the dye Fluo-4 in combination with structured illumination fluorescence microscopy: Representative microscopy images of the cells before and after the stimulation with the different treatments. From each experiment images with the maximum fluorescence signal after stimulation are shown. (a) Panel with the controls. (b) Panel with the nanoformulations. (c) Fluorescence intensity over time after stimulation with the different treatments (Scale bar = 200 μM). NC: nanocapsules (images in (a) and (b) have been contrast enhanced for better visualization in the print version).

Intracellular calcium fluxes are known to influence cell migration [64] and can also have an impact on cell morphology [40]. Thus, we loaded MDCK-C7 cells with fluo-4, a dye which emits fluorescence immediately after complexation with calcium ions, and recorded fluorescence microscopy images during their stimulation with the different treatments for 24 s. As a positive control, we used ionomycin, a compound which induces calcium transport through cellular membranes. **Fig 4**shows representative micrographs of the highest achieved fluorescence intensities for each measurement. The entire recorded image series are animated in video **S2 Movie** (Supporting Information). **Fig 4A** depicts the images for the controls and the treatment with free capsaicin. For the negative control, in which only the buffer was used, no fluorescence emission was detected. In contrast, in the positive control a sudden strong fluorescence signal was evident. In analogy to the negative control, the stimulation with 100 μM capsaicin induced no fluorescence signal. **Fig 4B** presents the results after the addition of unloaded and capsaicin-loaded nanocapsules. For both treatments fluorescence was detected. The time course for the measured fluorescence intensities of all treatments is plotted in **Fig 4C**. The positive control produces the strongest fluorescence signal immediately after stimulation. This response reaches a maximum after ~2 s and persists until the end of the observation period of ~24 s. Also, for the nanoformulations, an overall increase of the fluorescence intensity is detected, ~ 35% lower than for the positive control. In contrast to ionomycin, the observed effect is reversible (i.e., it peaks after ~5–10 s and fades after ~15–18 s). In the case of untreated cells and cells treated with free capsaicin, no fluorescence signal occurred during the experiment.

To gain further insight into the calcium influx results the presence of TRPV1 and TRPV4 receptor ionic channels in the cells was probed by polymerase chain reaction (PCR) (see supporting information **S1 File**). While the expression of TRPV4 was confirmed, the presence of TRPV1 could not be detected.

### Rearrangements of the cytoskeleton and tight junction proteins after treatment with encapsulated and free capsaicin

**Fig 5:**
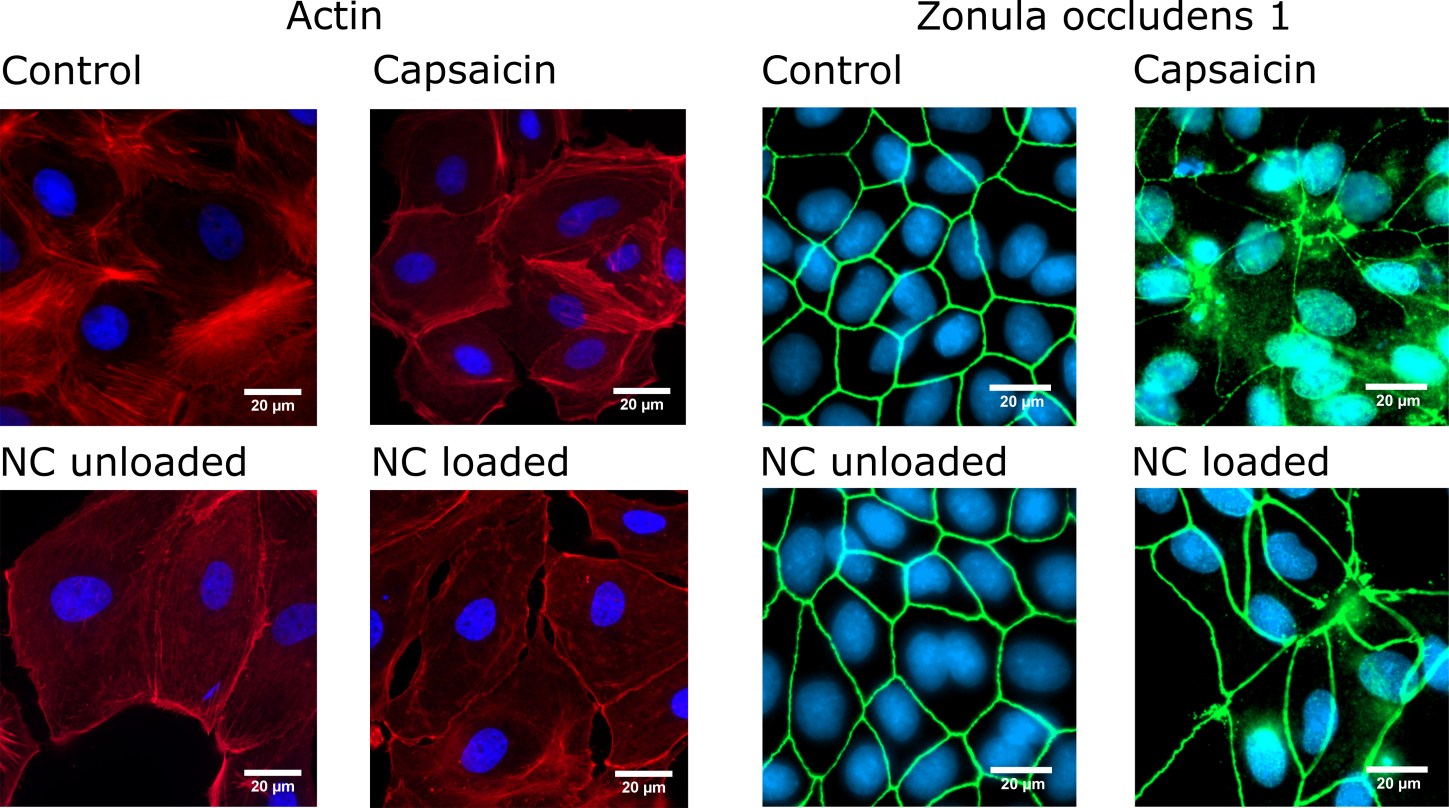
Fluorescence microscopy. Representative images of MDCK cells with actin or ZO-1 staining. (Nuclei: blue, ZO-1: green, Actin: red, NC: nanocapsules) (images have been contrast enhanced for better visualization in the print version)”.

To probe the influence of the chitosan nanocapsules on the cytoskeleton and the tight junctions we investigated changes in the actin filament structure and zonula occludens 1 (ZO-1) which is connected to many tight junction proteins and thus is a target to image tight junction protein rearrangements. In our previous studies [17] we have conducted time-course TEER measurements of cell monolayers and have shown that the highest ion permeability increase occurs at 6 hours. Based on these earlier findings, we have chosen this time point to investigate the tight junctions of the cell monolayer. The left panel of **Fig 5**shows representative fluorescence microscopy images of the actin distribution in the cells after exposure to the different treatments that also reflect their heterogeneous growth properties. The clearly visible filament structures for the control cells appear reduced after all treatments. In correspondence with the results in Fig 3 that were retrieved with DHM, the cells treated with unloaded and capsaicin-loaded nanocapsules show slightly more pronounced effects than the cells that were exposed to free capsaicin. Fluorescence of stained ZO-1 (right panel of **Fig 5**) showed continuous sharp lines along the cell-cell contacts for the control cells and the unloaded nanosystems. Notice that for the treatments with capsaicin and capsaicin-loaded nanocapsules, ZO-1 rearranges to discontinuous diffuse and disrupted structures with increased amounts of dislocated proteins in the cytosol and probably also in the nucleus. Note that a stronger effect is observed for free capsaicin than for the capsaicin-loaded nanoformulations which only cause few disturbances of the ZO-1 pattern. The distribution of the actin skeleton was further imaged for the loaded nanoformulation after 24 hours (see section 3 and **S1.3 Fig** of supporting information **S1 File**). No changes between the control and the treated sample were observed.

## Discussion

In this study, we have analyzed the migration behavior of MDCK-C7 cells after treatment with both, chitosan-based unloaded and capsaicin-loaded nanocapsules, in comparison to free capsaicin, and to untreated control cells. To this end, we recorded time-lapse micrographs by minimally-invasive observation with label-free DHM and analyzed the resulting quantitative phase images by cell tracking. This newly developed image analysis procedure quantifies global cell layer morphology changes from the interferometrically detected optical path length changes. Furthermore, we carried out investigations with fluorescence microscopy to gain insight into changes in the cytosolic calcium concentrations and to analyze rearrangements of the cytoskeleton and tight junction proteins. We found capsaicin and nanocapsules induced profound changes in the migration behavior of the cells. In general, all treatments induced increased cellular motility and speed and changed the migration from a mainly random pattern observed in the control, to a more directed, straight movements. The results from single-cell tracking reveal for all treatments that these effects were present over the entire time course of the experiments, as evidenced by the linear trends of the migration behaviors (**Fig 2D and 2E**). Error bars of similar magnitude support the reliability of the retrieved data. These results matched well with the averaged endpoint measurements (**Fig 2B and 2C**) where a higher migration speed corresponds to an increased cumulative migrated distance. The found increased directness of cell migration is also in agreement with increased values for the Euclidean distance. These findings also fit with the evaluation results of the DHM assay were global morphology changes of the observed monolayers (**Fig 3**) are correlated with rearrangements of the cytoskeleton and the distribution of tight junction proteins (**Fig 5**). In short, we found that changes of the migration behavior are accompanied by alterations of the global cell morphology in a time-dependent manner and rearrangements of actin and tight junction proteins as further discussed below.

Capsaicin is known to exert an effect on the migration behavior of HepG2 cells [43], which has been attributed to the influx of calcium into the cells mediated via the stimulation of the TRPV1 receptor. However, in the present study conducted on MDCK-C7 cells we were not able to detect a sudden influx of calcium into the cells right after the addition of capsaicin (**Fig 4**). Nevertheless, in general, the homeostasis of ions within the cell is also known to have an influence on cell motility [64], and the long-term calcium influx remains to be investigated. A PCR experiment showed TRPV1 was not expressed in the investigated cell line (see supporting information **S1 File**), in agreement with the lack of intracellular influx of calcium in the cells treated with free capsaicin. This result also suggests the observed effects of capsaicin on cell motility (**Fig 2**), might be governed by a different mechanism independent of calcium. Also, the cell morphology seemed to be affected (**Fig 3B**) after capsaicin and nanocapsules addition. The fact that the cell contours became clearly visible is an indicator for an increase of the height in the cell center which resulted in a more heterogeneous surface roughness (video **S1 Movie)**.

The increased phase values of the Y-intercepts of the DHM analysis of global morphology changes in **Fig 2**also indicate a fast change of the cell layer surface roughness within a few minutes shortly after the addition of the different treatments. Also, tight junction opening was clearly indicated in video **S1 Movie** which also had an impact on the morphology of the cell layer and is confirmed by impedance spectroscopy measurements of the TEER in our previous study [17]. This effect, caused by the disruption of the network of tight junctions, could enable the cells to move and migrate faster. Notice that the unloaded nanoformulation had a higher impact on the behavior than free capsaicin (**Fig 2**). This implies the nanoformulation itself must have had an impact on the cell motility which may stem from several possible reasons. One explanation could be the generation of a mechanical stimulus on the cell. It is known mechanical stimulation can lead to influx of calcium ions mediated via the TRPV4 receptor, which was proven to be expressed in the investigated cell line (supporting information **S1 File**). For endothelial cells, it has been shown mechanical stimulation had an impact on cell motility [65,66]. This would also correlate with our observation that the nanoformulation treatment produced a sudden calcium flux in the cells (**Fig 4**). Even though there was no change in calcium level visible directly after the initial influx it remains to be elucidated if there is a long-term effect on calcium influx related to this finding. The newly developed method to characterize global cell morphology changes using histograms and average phase values of quantitative DHM phase images concurs with the results from the motility analysis. Indeed, the Y-axis intercept of the average DHM phase contrast increased, which is consistent with an instant change of cell morphology. Also, the slope of the average DHM phase images indicated an increasing change in surface roughness of the cell layer after treatment with the nanocapsules (**Fig 3B and 3C**). In the time lapse video **S1 Movie** the cell contours are clearly visible which also indicates a change in surface roughness. The highest values for migration speed and directness were observed for the loaded nanocapsules (**Fig 2**) which is probably the result of the combined effect of capsaicin and the nanosystem. The DHM morphology assay showed here the highest increase in surface roughness concurring with the highest Y-axis intercept and slope (**Fig 3B and 3C**). In the corresponding time lapse video **S1 Movie**, it was also visible that tight junctions opening was more pronounced compared to the unloaded system. The observed weakening of the entire cellular network mediated by TJs and the actin skeleton could be an explanation for the overall increase in speed of displacement (**Fig 5**). Indeed, a recent study has revealed interference with a connexin 43/ZO-1 complex regulates cerebral endothelial F-actin architecture and directed cell migration [67]. Antibody-mediated perturbation of the connexin 43/ZO-1 complex resulted in disruption of actin filaments, increased migration speed, and increased directionality of migration. In this respect, the study shows a striking similarity to our findings in capsaicin- and loaded nanocapsule-treated cells, thus suggesting a potentially similar mechanism of action. Regarding directional and collective cell migration, several mechanisms have been discussed, including spatially constrained ERK activation in leader cells, contact guidance, haptotaxis and chemotaxis [68]. At least some of these mechanisms could have contributed to the effect exerted by the capsaicin or loaded nanocapsule treatments. For example, (modified) chitosans in general are capable of accelerating cell migration in wound healing [69], and have a known effect on the ERK signaling pathway which has been shown previously [70]. Moreover, capsaicin was recently shown to affect integrin function [71], which may have an impact on collective haptotactic cell migration. While further work is needed to elucidate the detailed molecular mechanism underpinning the observed influence of capsaicin or loaded nanocapsule treatments, our study provides a basis for future unfolding mechanistic investigations.

## Conclusions

In summary, the treatment of MDCK-C7 cells with capsaicin encapsulated by a chitosan-coated nanosystem and in its free form resulted in a change of the cellular morphology as well as velocity and directness of migration compared to control cells while also the unloaded system demonstrated a change in these properties in a very similar way. Calcium influx occurred for the nanoformulations, but not for free capsaicin, which suggests an alternative mechanism could be at play as the TRPV1 receptor was not expressed in the investigated MDCK-C7 cell line. A newly developed DHM assay, that quantifies cell layer surface roughness changes by interferometric detection of cell induced optical path length changes, afforded complementary quantitative information about the global morphological alteration of the cells that are accompanied with the findings from cell tracking and calcium influx measurements. Protein redistributions within the cells (actin, ZO-1) revealed by fluorescence microscopy further support the presence of cell morphology changes. Additional details of the underlying mechanisms at molecular level governing the stimulation of epithelial cells with nanoencapsulated and free capsaicin that induce their motility and change in morphology remain to be elucidated. “Why do the stimulated cells move in an orchestrated manner and the same direction?” is a question that needs to be answered. Chemotaxis induced by local gradients of concentration of nanocapsules as well as other unknown effects, cannot be ruled out, whereas the disruption of ZO-1 function and the associated restructuring of the actin cytoskeleton may provide some clues. It also remains to be discovered what is the significance of these phenomena for single cells, groups of cells and *in vivo*. The developed nanosystem is a potential new drug carrier for hydrophobic drugs due to the tight junction opening abilities which could also be used for functional food applications as it is only comprised of materials of natural origin. Ongoing studies in our groups investigate the feasibility of the system as a gene transfection agent with promising results.

## Supporting information

S1 Movie Dynamic imaging of MDCK-C7 cell with quantitative digital holographic phase contrast. Movie of quantitative phase images resulting from time-lapse observations of MDCK-C7 cells with digital holographic microscopy after treatment with free capsaicin, unloaded chitosan nanocapsules and capsaicin-loaded chitosan nanocapsules in comparison to untreated control cells.
(WMV)

S2 Movie Temporal development of calcium flux. Movie of temporal development of calcium flux utilizing the dye Fluo-4 as supporting information for Fig 4.
(WMV)

S1 File Supporting information about cell viability, presents of TRPV1 and TRPV4 and actin distribution. Supporting information about cell viability of MDCK-C7 cells after treatment with free capsaicin for DHM time-lapse observation conditions, ribonucleic acid (RNA) isolation and polymerase chain reaction (PCR) for probing the presents of TRPV1 and TRPV4, and fluorescence microscopy of actin in MDCK-C7 cells 24 hours after treatment with capsaicin loaded chitosan nanocapsules. S1_File.pdf).
(PDF)
