# Supplementary material for: Nanoencapsulated capsaicin changes migration behavior and morphology of madin darby canine kidney cell monolayers
Source: PLoS One. 2017 Nov 6;12(11):e0187497. doi: 10.1371/journal.pone.0187497 (PMC5673207; doi:10.1371/journal.pone.0187497)
Supplement: S1 File — Supporting information about cell viability of MDCK-C7 cells after treatment with free capsaicin for DHM time-lapse observation conditions, ribonucleic acid (RNA) isolation and polymerase chain reaction (PCR) for probing the presents of TRPV1 and TRPV4, and fluorescence microscopy of actin in MDCK-C7 cells 24 hours after treatment with capsaicin loaded chitosan nanocapsules. S1_File.pdf). (PDF) [file pone.0187497.s003.pdf]

Supplementary information S1 File

Encapsulated Capsaicin Changes Migration Behavior and Morphology of Madin Darby Canine Kidney Cell Monolayers

Mathias Kaiser[1], Luisa Pohl[2], Steffi Ketelhut[2], Lena Kastl[2], Christian Gorzelanny[3], Martin Götte[4], Jürgen Schnekenburger[2], Francisco M. Goycoolea[1,5 **], Björn Kemper[2*]

[1] Institute of Plant Biology and Biotechnology (IBBP), Westfälische Wilhelms-Universität Münster, Schlossgarten 3, Münster 48149, Germany

[2] Biomedical Technology Center of the Medical Faculty, Westfälische Wilhelms-Universität Münster, Mendelstraße 17, Münster 48149, Germany

[3] Experimental Dermatology, Department of Dermatology, Medical Faculty Mannheim, Heidelberg University, Theodor-Kutzer-Ufer 1-3, Mannheim 68167, Germany

[4] Department of Gynecology and Obstetrics, Westfälische Wilhelms-Universität Münster, Albert-Schweitzer-Campus 1, Münster 48149, Germany

[5] School of Food Science & Nutrition, University of Leeds, Leeds LS2 9TJ, UK

Corresponding authors:

E-mail: **goycoole@uni-muenster.de.

E-mail: *bkemper@uni-muenster.de.

1. Cell viability of MDCK-C7 cells after treatment with free capsaicin using DHM conditions

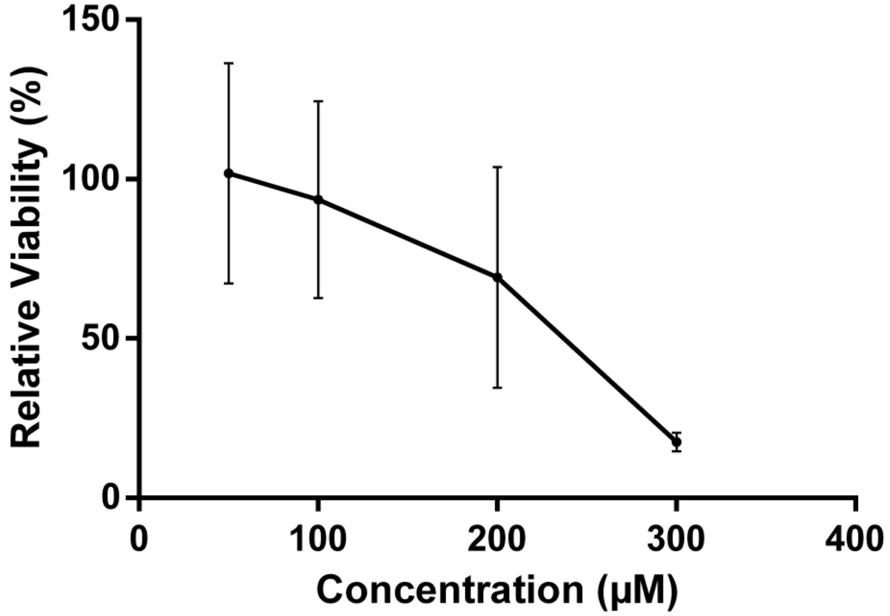

Fig S1.1. Cell Viability of MDCK-C7 cells after treatment with free capsaicin using conditions for observation with digital holographic microscopy (DHM): No CO_2 enriched atmosphere, HEPES buffer in medium. MTT assay for cell viability: The experiment shows that the cytotoxic effect occurs at a concentration of 200 µM under the investigated conditions.

Mandin Darby Canine Kidney (MDCK) cells clone C7 were cultured in 75 cm^2 flasks using MEM supplemented with 10% fetal bovine serum, 1% L-glutamine (200 mM) and 1% penicillin-streptomycin (10000 units penicillin, 10000 units streptomycin in 0.9% NaCl). The cultures were maintained in a humid atmosphere at 37°C with 5% CO_2 (Sanyo MCO-19AIC, Panasonic Bio-medical Sales Europe BV, AZ Etten Leur, Netherlands). Cells from passages 22–34 were used for all experiments, which were carried out as independent triplicates on different days. After reaching microscopic confluence, the cells were washed with 10 ml phosphate buffered saline (PBS) and trypsinized with 10 ml 0.05 % trypsin in EDTA (1x) buffer. After detachment, 10 ml of MEM was added to the trypsin buffer. The cell suspension was centrifuged at 1000 rpm for 5 min (Rotina 420 R, Hettich GmbH, Tuttlingen, Germany). The excess of medium was removed and the cell pellet was resuspended in 1 ml MEM. A 10-µl aliquot of the cell suspension was diluted

with 90 µl trypan blue and the number of cells was counted with an improved Neubauer chamber before seeding. The cells were subcultured by splitting at a ratio of 1:10.

The cytotoxicity of capsaicin was evaluated using an MTT assay. Briefly, 100 µl of cell suspension was transferred to each well of a 96-well tissue culture plate (~104 cells per well or ~105 cells/ml) and allowed to attach for 24 h. The cells were washed twice with supplement-free MEM before the sample was added. The cells were incubated with the sample for 24 h without a CO_2 enriched atmosphere at 37 °C using supplement-free MEM with HEPES buffer (20 mM). The samples were removed and replaced with 100 µl supplement-free MEM and then again cultured in regular conditions. We prepared an MTT solution in PBS with a concentration of 5 mg/ml of thiazolyl blue tetrazolium bromide and added 25 µl to each well. After 4 h, the medium was again removed and the dye was dissolved in DMSO. After orbital shaking at 300 rpm for 15 min, the absorbance was measured at λ = 570 nm in a microplate reader (Safire, Tecan AG, Salzburg, Austria). Relative viability values were calculated by dividing individual viabilities by the mean of the control. We used 4% Triton X-100 in PBS as a positive control.

2. Ribonucleic acid (RNA) isolation and polymerase chain reaction (PCR) for probing the presents of TRPV1 and TRPV4

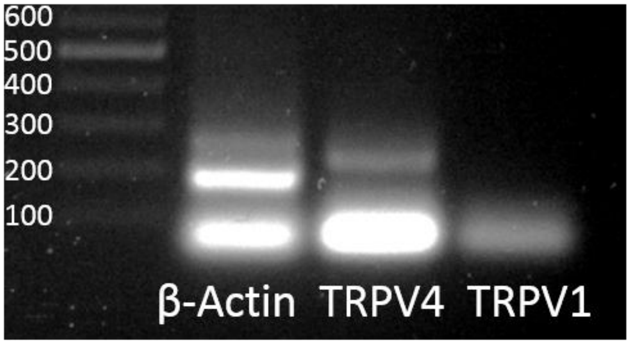

Fig S1.2. PCR products in in a 1% w/v agarose gel. While the bands for β-actin and TRPV4 are visible a band for TRPV1 was not observed.

Total RNA was extracted using the RNeasy mini kit (Qiagen, Hilden, Germany), following the manufacturer's protocol. The cDNAs were synthesized with the reverse transcription system (Promega, Madison, USA) from 1 µg of total RNA per sample by following the manufacturer's instructions. The PCR primer sets were designed as follows: TRPV1 canis Forward: 5'- CCCCTGGATGGAGACCCTAA -3' and Reverse, 5'- ATGGCAATATGCAGGGCTG -3'; TRPV4 canis Forward, 5'- GATCGGGGTCTTTCAGCACA -3' and Reverse, 5'- TCCCGCAGCAGTTCATTGAT -3'; β-actin canis Forward, 5'- CAAAGCCAACCGTGAGAAG -3' and Reverse, 5'- CAGAGTCCATGACAATACCAG -3'. The primer sets were purchased from Eurofins MWG Operon (Ebersberg, Germany). Amplification was performed using a Thermocycler of the type C1000 (Bio-Rad Laboratories GmbH, Munich, Germany). Cycling conditions were 95°C for 2 min, and then 40 cycles at 95°C for 15 s and 60°C for 1 min. The PCR products were ran for 40 minutes in a 1% w/v agarose gel. A DNA ladder was also included (GeneRuler™ DNA Ladder Mix, Thermo Fisher Scientific, Waltham, USA).

3. Fluorescence microscopy of actin in MDCK cells 24 hours after treatment with capsaicin loaded chitosan nanocapsules

Control NC loaded

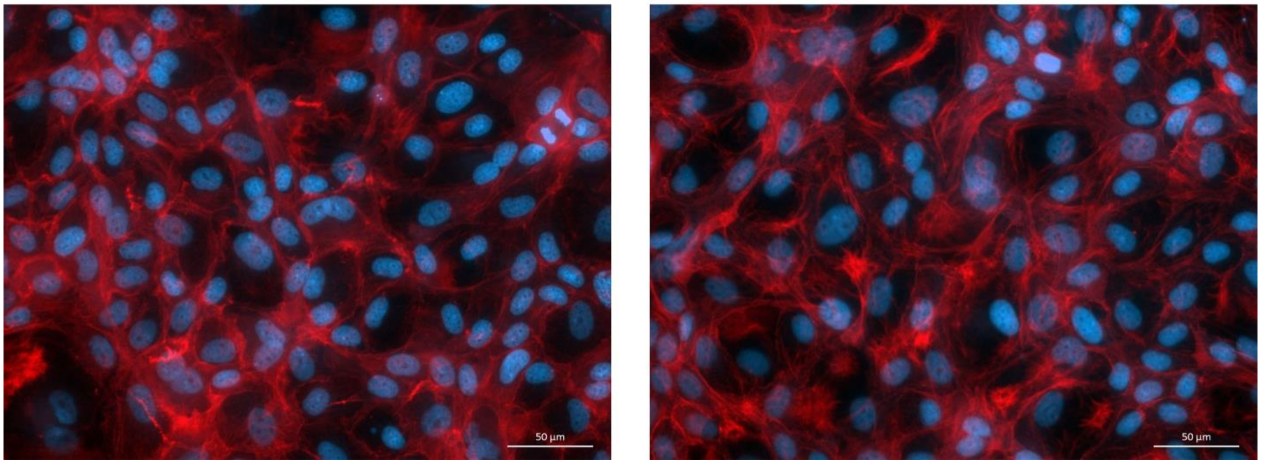

Fig S1.3. Fluorescence imaging of actin in MDCK cells 24 hours after treatment with capsaicin loaded chitosan nanocapsules (ratio 1:40) in comparison to an untreated control. (nuclei: blue, actin: red, NC: nanocapsules). Cells were stained with phalloidin and DAPI as described in section “Fluorescence microscopy of actin and ZO-1” in the main document (images have been contrast enhanced for improved visualization).
